# The BPH/5 Mouse Model of Superimposed Preeclampsia Is Not a Model of HELLP Syndrome

**DOI:** 10.3390/biology10111179

**Published:** 2021-11-14

**Authors:** Andrea N. Johnston, Tifini L. Batts, Ingeborg M. Langohr, Cambri Moeller, Chin-Chi Liu, Jennifer L. Sones

**Affiliations:** 1 Departments of Veterinary Clinical Sciences, School of Veterinary Medicine, Louisiana State University, Baton Rouge, LA 70803, USA; batts1@lsu.edu (T.L.B.); cmoeller1@lsu.edu (C.M.); cliu@lsu.edu (C.-C.L.); jsones@lsu.edu (J.L.S.); 2 Pathobiological Sciences, School of Veterinary Medicine, Louisiana State University, Baton Rouge, LA 70803, USA; ilangohr@lsu.edu

**Keywords:** preeclampsia, HELLP syndrome, hepatic steatosis, BPH/5

## Abstract

**Simple Summary:**

HELLP syndrome ((H) for hemolysis, (EL) for elevated liver transaminases, and (LP) for low platelets) occurs in up to 20% of women with severe preeclampsia. Disrupted placental development is causal to both preeclampsia and HELLP syndrome, yet why HELLP syndrome develops in some women remains unclear. Targeted treatments for this devastating disease are currently unavailable, and the development of preclinical models is imperative. Therefore, we sought to determine whether the blood pressure high subline 5 (BPH/5) mouse, a spontaneous model of preeclampsia, could also serve as a model of HELLP syndrome. Although anemia, thrombocytopenia, and plasma markers of liver dysfunction were not found in the BPH/5 mouse during pregnancy, precluding it as a model of PE associated with HELLP syndrome, a progressive fatty liver phenotype was identified. The BPH/5 mouse may be useful as a model of hepatic steatosis in superimposed preeclampsia.

**Abstract:**

Preeclampsia (PE) is a multisystemic disease of pregnancy affecting 2–8% of women worldwide. PE-induced liver disease is a rare but important complication of pregnancy. The pathogenesis of liver dysfunction in PE is poorly understood, but is correlated with dysregulated angiogenic, inflammatory, and hypoxic events in the early phase of placental development. Because BPH/5 mice develop the maternal and fetal hallmarks of PE during pregnancy, we hypothesized that they may also share the clinicopathologic findings of the human PE-associated hemolysis elevated liver transaminases low platelets (HELLP) syndrome. Using this model, we determined that microangiopathic hemolysis, thrombocytopenia, and elevated liver enzymes do not occur in mid to late gestation. Pregnant BPH/5 mice do not develop histologic evidence of hepatic inflammation, but they do have increased microsteatosis scores at preconception and in mid to late gestation that progress to macrosteatosis in a subset of mice in late gestation. The transcriptional upregulation of TNF-α, CXCL-10, and TLR-2 occurs in mid gestation prior to the onset of macrosteatosis. The BPH/5 female mouse is not a model of HELLP syndrome, but may be a model of fatty liver disease associated with pregnancy.

## 1. Introduction

Preeclampsia (PE) affects up to 8% of all pregnant women [1]. Hemolysis, elevated liver transaminases, and low platelets (HELLP) syndrome occurs in 0.2–0.6% of all pregnancies and in up to 20% of women with severe PE [2,3]. The pathogenesis of liver dysfunction in PE and HELLP syndrome are poorly understood. Dysregulated angiogenesis in early pregnancy leading to endothelial injury and maternal–fetal metabolic irregularities contribute to the pathogenesis of both syndromes [4,5,6,7,8]. In HELLP syndrome, secondary hepatic microangiopathy results in a proinflammatory cascade promoting sinusoidal occlusion and hepatocellular necrosis [9,10]. The diagnosis of HELLP relies predominantly on clinical signs and liver function tests as biopsies of the liver are risky in late gestation. Two classification systems, the Tennessee Classification System and the Mississippi-Triple Class System, are used to identify HELLP syndrome in women [11]. Both systems identify hemolysis based on a peripheral blood smear, increased serum bilirubin, and an elevated lactate dehydrogenase (LDH). Liver injury is quantified by hepatic transaminase elevation (aspartate or alanine aminotransferase (AST, ALT)), and a platelet count identifies the degree of thrombocytopenia. The Mississippi-Triple Class System subclassifies severity based on platelet nadir and AST/ALT elevation.

The blood pressure high subline 5 (BPH/5) mouse was first described as a model of PE in 2002 [12]. Since that time, this model has been extensively characterized [13,14,15,16,17,18,19,20]. The preconception BPH/5 female is borderline hypertensive with increased subcutaneous and visceral adiposity and dyslipidemia [19,21,22]. This phenotype predisposes to the spontaneous development of the superimposed PE-like syndrome, which manifests with hypertension, proteinuria, and reduced fetal viability [12,19]. In early pregnancy, BPH/5 mice exhibit placental dysfunction resulting in compromised maternal–fetal circulation and a progressive inflammatory response at the utero–placental interface [17,18,19,23]. A combination of factors influence the development of secondary systemic effects including liver injury. Because BPH/5 mice develop the maternal and fetal hallmarks of PE during pregnancy, we hypothesized that they may also share the clinicopathologic findings of human PE-associated HELLP syndrome. A spontaneous murine model of HELLP syndrome has not been identified. Because such a model would help to characterize the pathophysiology of the human HELLP syndrome, we sought to determine whether pregnant BPH/5 shared clinical features of the syndrome. Using this model, we determined whether evidence of microangiopathic hemolysis, thrombocytopenia, or elevated liver enzymes occur in mid to late gestation and tested the hypothesis that the BPH/5 mice develop histologically evident hepatic injury and transcriptional upregulation of inflammatory mediators.

## 2. Materials and Methods

### 2.1. Animals and Husbandry

BPH/5 mice aged 8 to 12 weeks old were housed together with ad libitum access to standard chow in an environment with a 12 h light-dark cycle. All animal use procedures were approved by the Louisiana State University School of Veterinary Medicine Animal Care and Use Committee. Care of the mice was in accordance with the National Institute of Health’s guide for the Care and Use of Laboratory Animals and the American Veterinary Medical Association Panel on Euthanasia.

### 2.2. Mouse Experiments

Timed pregnancies were established in 22 BPH/5 female mice (1 male and 1 female per cage). Male mice were removed after detection of a copulatory plug, and visualization was designated as embryonic day 0.5 (e0.5). Females were euthanized with CO_2_ at day e12.5 (*n* = 8) and e18.5 (*n* = 14) of pregnancy. These time points were chosen to represent the human gestation periods of mid to late gestation. Fifteen 8–12-week-old non-pregnant (NP) BPH/5 females were euthanized to serve as controls. Blood, obtained by cardiac puncture, and liver were collected following euthanasia.

### 2.3. Clinicopathologic Parameters

A blood smear was prepared using whole blood for the evaluation of red blood cell morphology and platelet count. The remaining blood was aliquoted to a heparinized microhematocrit tube and a 1-milliliter lithium heparinized gel separator tube. The microhematocrit tube was centrifuged for 3 min in a BD Clay Adams™ TRIAC^®^ centrifuge, and the packed cell volume was immediately determined. Approximately 500 µL of plasma was collected following centrifugation at 1000× *g* for 15 min. Plasma was stored at −80 °C until the biochemical analysis was completed. The Louisiana State University’s Veterinary Clinical Pathology Service measured albumin, AST, blood urea nitrogen (BUN), and total bilirubin on a Beckman AU680 Clinical Chemistry Analyzer according to the manufacturer’s protocols. Lactate dehydrogenase (LDH) was measured using a colorimetric assay kit from Abcam™ (ab102526).

### 2.4. Liver Histology

The liver was sectioned, and samples were immediately fixed in 10% neutral buffered formalin for 24 h prior to paraffin embedding and being flash frozen in liquid nitrogen prior to storage at −80 °C. The Louisiana Animal Disease and Diagnostic Lab processed the formalin-fixed samples. Hematoxylin & Eosin (H&E) staining was performed on all liver samples to evaluate hepatic histomorphology. Histology samples were scored for steatosis and inflammation by a blinded board-certified veterinary pathologist (IML) using the scoring system by Liang et al. [24].

### 2.5. Quantitative Reverse Transcription PCR

The total RNA was harvested from a flash-frozen liver using the Qiagen^®^ RNA extraction kit. cDNA was synthesized, and relative gene expression was determined by quantitative (q)RT-PCR using the SYBR Green (Qiagen) as previously described [25]. Gene expression was assessed using standard qPCR approaches with the iTaq™ universal SYBR^®^ Green Supermix. An analysis was performed on the 7900 HT Applied BioSystems™ Real-Time PCR Detection System (BioRad, Bedford, MA, United States). All primer sequences are listed in Appendix A. Each quantitative RT-PCR was performed in duplicate with 12.5 ng cDNA. The relative expression levels of the target genes were calculated and expressed as ddCt relative to 18S RNA expression in NP BPH/5 mice [26].

### 2.6. Statistical Analyses

The data are presented as a mean ± standard deviation (parametric) or median and interquartile range, IQR, (nonparametric). The descriptive statistics of each continuous variable were determined for each cohort. An ANOVA with Fisher’s LSD post hoc test or the Kruskal–Wallis test was performed when the data were normally distributed or not normally distributed, respectively. The value of *p* < 0.05 was considered statistically significant. The analysis was completed using the JMP Pro 15 (SAS Institute Inc., Cary, NC, USA). Graphs were generated using the GraphPad Prism, Version 9.1.0 (GraphPad Software, San Diego, CA, USA).

## 3. Results

### 3.1. Clinicopathologic Parameters

**Figure 1:**
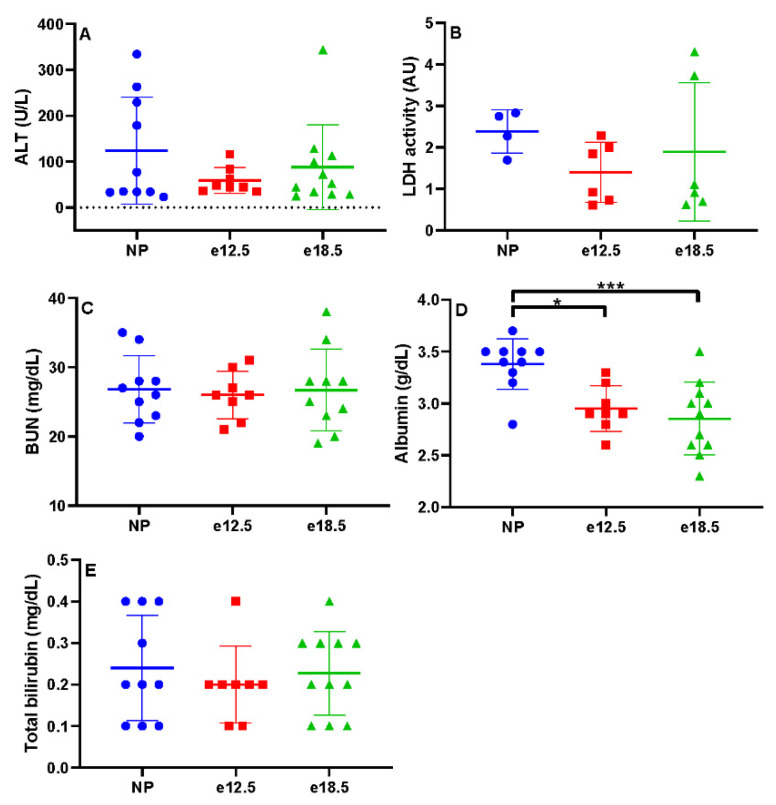
Biochemical markers of liver disease in NP, e12.5, and e18.5 BPH/5 mice (*n* = 8–15 per group). There was no significant difference in plasma levels of (**A**) alanine amino transferase (ALT), (**B**) lactate dehydrogenase (LDH), (**C**) blood urea nitrogen (BUN), (**D**) albumin, and total bilirubin (**E**) at any time point. * *p* < 0.01, *** *p* < 0.001, ns, not significant.

There were no significant differences in LDH, ALT, BUN, or total bilirubin between groups (Figure 1). The plasma albumin levels in BPH/5 mice at mid (2.95 g/dL) and late (2.86 g/dL) gestation were significantly decreased from NP BPH/5 mice (3.38 g/dL; *p* < 0.01, *p* < 0.001, respectively).

**Figure 2:**
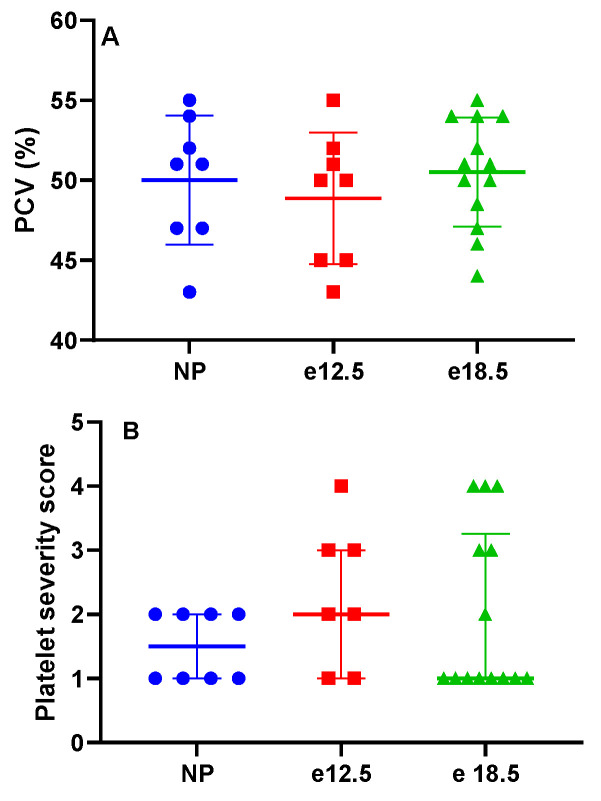
Clinicopathologic markers in NP, e12.5, and e18.5 BPH/5 mice (*n* = 8–15 per group). There was no evidence of (**A**) anemia or (**B**) progressive thrombocytopenia during pregnancy. Packed cell volume (PCV).

There was no significant difference in packed cell volume or platelet count during pregnancy (Figure 2). There was no evidence of microangiopathic red blood cell injury on blood smears.

### 3.2. Liver Histology

**Figure 3:**
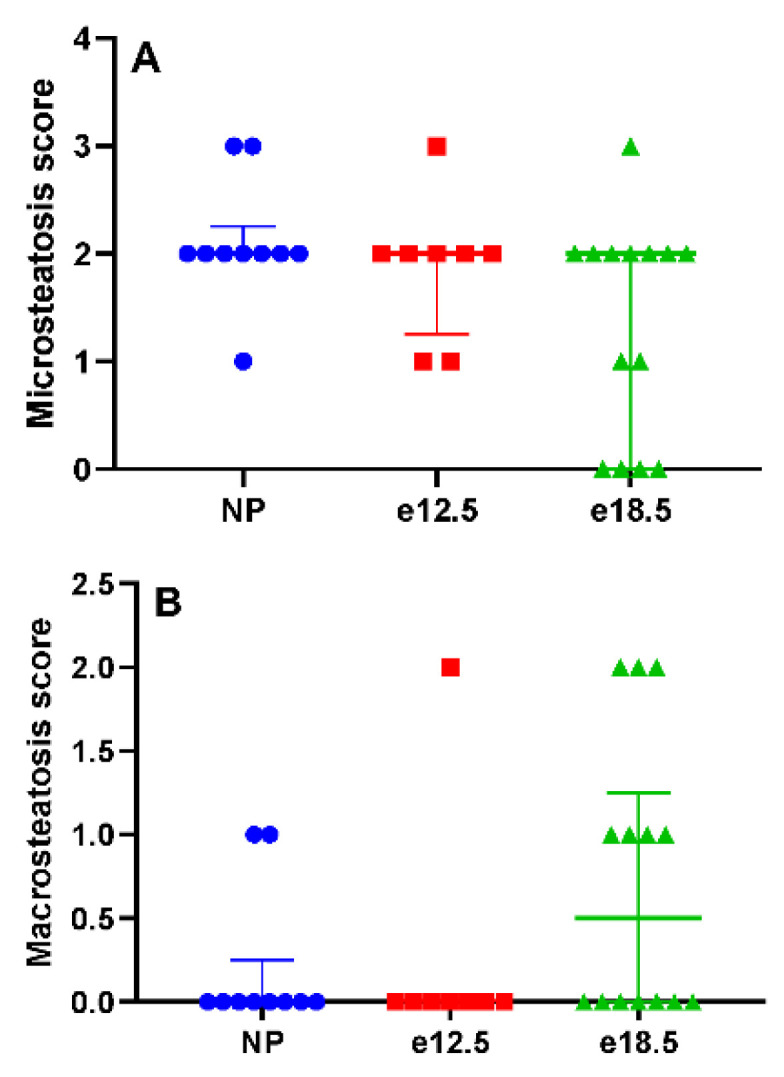
Histologic hepatic steatosis scores in NP, e12.5, and e18.5 BPH/5 mice (*n* = 8–15 per group). (**A**) A score of 0 equates to no microsteatosis observed, and 3 is severe. Microsteatosis was observed at all time points. There was no significant difference between groups. (**B**) A score of 0 equates to no macrosteatosis observed, and 3 is severe. Macrosteatosis scores increased in a subset of mice at e18.5. There was no significant difference between groups.

Histologically, there was no evidence of significant necroinflammatory liver disease at any time point before or during pregnancy. Inflammation was scored on a scale from 0 (no inflammatory infiltrate) to 3 (severe inflammation). NP BPH/5 had a mean score of 0, e12.5 mice had a mean score of 0.125, and e18.5 mice had a mean score of 0. The mean hepatic microsteatosis score was elevated at all time points (Figure 3A), and no significant difference was detected (*p* < 0.05). NP BPH/5 had a median score of 2 (IQR: 2–2.25), e12.5 mice had a median score of 2 (IQR: 1.25–2), and e18.5 mice had a median score of 2 (IQR: 0–2). The mean hepatic macrosteatosis score was elevated in late gestation (Figure 3B), and no significant difference was detected (*p* < 0.05). NP BPH/5 had a median score of 0 (IQR: 0–0.25), e12.5 mice had a median score of 0, and e18.5 mice had a median score of 0.5 (IQR: 0–1.25).

### 3.3. Transcriptional Markers of Inflammation

**Figure 4:**
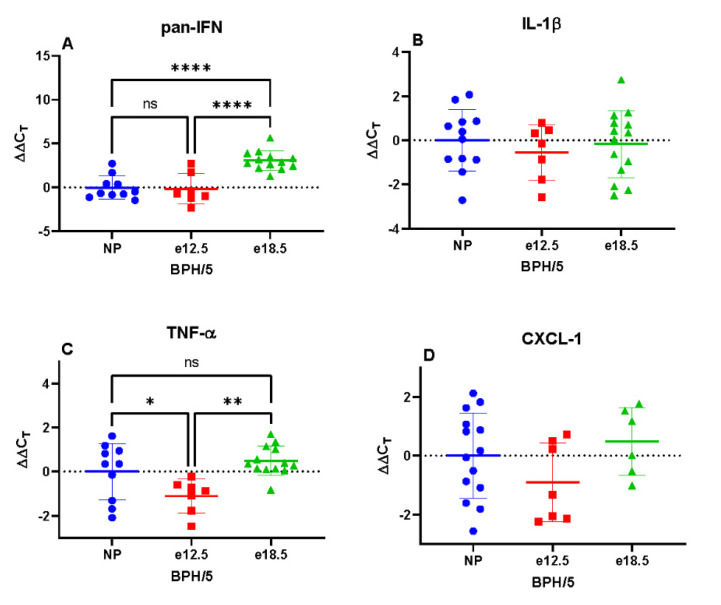

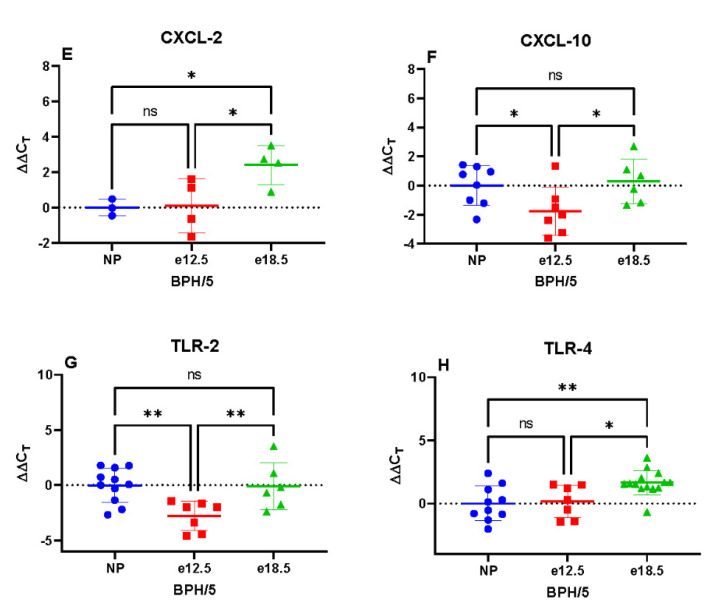
Transcriptional expression of hepatic inflammatory markers in BPH/5 mice preconception and at mid-late gestation (*n* = 7–15 per group). (**A**) Pan interferon (IFN) mRNA expression was significantly downregulated at e18.5, (**B**) Interleukin 1-beta (IL-1β) was unchanged during gestation, (**C**) Tumor necrosis factor-alpha (TNFα) expression was upregulated at e12.5, (**D**) C-X-C motif chemokine ligand-1 (CXCL-1) was unchanged during gestation, (**E**) C-X-C motif chemokine ligand-2 (CXCL-2) mRNA expression was significantly downregulated at e18.5, (**F**) C-X-C motif chemokine ligand-10 (CXCL-10) mRNA expression was significantly upregulated at e12.5, (**G**) Toll-like receptor-2 (TLR-2) transcription was significantly upregulated at e12.5, (**H**) Toll-like receptor-4 (TLR-4) expression was significantly downregulated at e18.5. * *p* < 0.05, ** *p* < 0.01, **** *p* < 0.0001 ns, not significant.

BPH/5 e12.5 mice had a significantly increased mRNA expression of TNFα, CXCL-10, and TLR-2 compared to NP and e18.5 mice. Hepatic IFN, CXCL-2, and TLR-4 mRNA expression was significantly reduced at e18.5 compared to preconception and e12.5 time points. There was no significant difference in IL-1β and CXCL-1 between groups (Figure 4).

## 4. Discussion

Approximately 10–20% of preeclamptic pregnancies have concurrent HELLP syndrome. Few models are available to study both adverse pregnancy outcomes simultaneously. Mice lacking eNOS given adenoviral sFlt1 exhibit hepatic dysfunction similar to what is observed in HELLP syndrome, but are unable to achieve pregnancy [27]. The widely used reduced uterine perfusion pressure (RUPP) rat model of preeclampsia was found to not have characteristics of HELLP [28] (Isler et al. 2003). When pregnant rats are given sFlt1 and soluble endoglin at mid gestation, it has been shown to mimic key features of severe preeclampsia with HELLP [9]. However, identifying animal models that spontaneously develop both preeclampsia and HELLP would allow for preconception interventions and early pregnancy diagnoses to be studied.

BPH/5 female mice have been used for several decades to investigate the pathogenesis of PE. These mice are obese with borderline hypertension before pregnancy, which is exaggerated by mid gestation, making them a model of superimposed PE. Besides maternal hypertension, this inbred genetic model exhibits other key features of PE. BPH/5 female mice are known to have an onset of renal disease during pregnancy, whereas the clinical pathologic demonstration of hepatic dysfunction or HELLP syndrome has yet to be described. While BPH/5 female mice do have elevations in circulating sFlt1 in mid gestation [21], soluble endoglin has not been measured, and the findings within would suggest they don’t have elevations in soluble endoglin as HELLP features were not observed in BPH/5 pregnant mice. This is important to evaluate given that BPH/5 female mice have obesity and dyslipidemia before and during pregnancy, and the pregnancy-induced changes described herein are not associated with HELLP syndrome. Herein, we investigated for the first time whether the BPH/5 mouse model of PE exhibits key features of HELLP syndrome during pregnancy.

Compared to clinical pathological parameters before pregnancy, liver biomarkers (ALT, LDH, BUN, and total bilirubin) were unchanged in mid and late gestation in BPH/5 pregnancies. Furthermore, PCV and platelets remained at consistent levels throughout pregnancy. However, plasma albumin progressively decreased in BPH/5 females during pregnancy. The absence of anemia, thrombocytopenia, and plasma markers of hepatic dysfunction precludes the BPH/5 mouse as a model of PE-associated HELLP syndrome. The progressive plasma hypoalbuminemia is likely secondary to the previously reported proteinuria in the BPH/5 model correlated with renal glomerular sclerosis identified histologically [12]. A high catabolic rate may be contributing to the significant reduction in plasma albumin as well [23]. BPH/5 female mice have increased white adipose tissue, which does not mobilize with pregnancy to meet the increased energy demand of pregnancy [21]. Interestingly, hepatic microsteatosis was identified prior to and during pregnancy in the BPH/5 female mice, and macrosteatosis developed in late gestation in over half of the mice. Despite the metabolic demands of late pregnancy, there is a shift in hepatic lipid mobilization to a fatty liver phenotype that may be detrimental to dam and offspring. The contribution of lean mass to the metabolic profile of BPH/5 female mice before and during pregnancy is unknown. Additional work will be required to assess hepatic metabolic function in the BPH/5 PE-like mouse model.

While histologically there was no significant evidence of inflammatory infiltrates in the liver at any time point, there were changes in proinflammatory signal transcription during the course of pregnancy. The upregulation of mid-gestational hepatic TNFα may be secondary to the previously described early gestation uterine proinflammatory state in BPH/5 mice; however, other systemic processes such as dyslipidemia may be contributing to it, too. TLR-2 and CXCL-10 mRNA were also upregulated at e12.5. TLR-2, a myeloid plasma membrane pathogen recognition receptor, is stimulated by a diverse array of pathogen-associated molecular patterns and may reflect gastrointestinal dysbiosis or portal vascular clearance of damaged cellular components [29]. The chemokine CXCL-10 is secreted by a variety of cells including monocytes, endothelial cells, and fibroblasts [30]. Because total hepatic RNA was extracted, it is uncertain which cell type is upregulating CXCL-10 transcription; regardless, CXCL-10 signaling is associated with T-helper activation and the magnification of a proinflammatory signaling cascade. The relative decrease in IFN, TLR-4, and CXCL-2 at late gestation is undetermined. A comparison with other mouse strains will be needed to determine whether the expression of these inflammatory markers differs in normal gestation.

Although PE and HELLP share clinical features, evidence suggests that the etiology and the clinical presentation of each syndrome are distinct [11,31,32]. Up to 20% of HELLP patients do not have hypertension or proteinuria, which are hallmarks of PE. The etiologies of PE and HELLP syndrome are both thought to include abnormal placental development leading to a placental antiangiogenic factor released into the maternal circulation [8]. Yet, differences in cytokine expression and angiogenic profiles are reported [33,34,35]. For example, HELLP patients without PE have significantly lower sFlt-1/PlGF ratios (soluble fms-like tyrosine kinase-1/placental growth factor) than gestational age-matched patients with PE or PE and HELLP [36]. BPH/5 female mice exhibit a profound angiogenic imbalance with increased circulating sFlt-1 and decreased VEGF and PlGF [20,21]. Therefore, BPH/5 mice without clinical pathologic features of HELLP represent the population of women that have PE without HELLP signs.

## 5. Conclusions

Ongoing research on HELLP syndrome suggests that it is a syndrome distinct from but coinciding with PE. Animal models of HELLP syndrome should be considered when investigating this pregnancy-related syndrome. Models of PE should not be assumed to exhibit this distinct and important subset of adverse pregnancy outcomes seen with HELLP syndrome.

## Data Availability

Datasets can be requested from the corresponding author.

## Appendix A

**Table A1:** Sequences of forward and reverse primers used in quantitative RT-PCR.

Primers	qPCR Primer Sequences (5′→3′)
Pan Interferon (pan-IFN)	CAACCCTCCTAGACTCATTCTGCA
	TATTTCCTCACAGCCAGCAG
Interleukin 1 beta (IL-1β)	TCCAGAGTGTGGATCCCAAGCAAT
	TGTCCTGACCACTGTTGTTTCCCA
Tumor necrosis factor alpha (TNF-α)	AGCCGATGGGTTGTACCTTGTTCTA
	TGAGATAGCAAATCGGCTGACGGT
C-X-C motif chemokine ligand-1 (CXCL-1)	TGAGCTGCGCTGTCAGTGCCT
	AGAGCCAGCGTTCACCAGA
C-X-C motif chemokine ligand-2 (CXCL-2)	GCTGGCCACCAACCACCAGG
	AGCGAGGCACATCAGGTACG
C-X-C motif chemokine ligand-10 (CXCL-10	ACCATGAACCCAAGTGCTGCCGTC
	GCTTCACTCCAGTTAAGGAGCCCT
Toll like receptor 2 (TLR-2)	TGCTTTCCTGCTGGAGATTT
	TGTAACGCAACAGCTTCAGG
Toll like receptor 4 (TLR-4)	ACCTGGCTGGTTTACACGTC
	CTGCCAGAGACATTGCAGAA
18s RNA (18s)	GTAACCCGTTGAACCCCATT
	CCATCCAATCGGTAGTAGCG

## References

[1] Ghulmiyyah L., Sibai B. (2012). Maternal Mortality from Preeclampsia/Eclampsia. Semin. Perinatol..

[2] Benedetto C., Marozio L., Tancredi A., Picardo E., Nardolillo P., Tavella A.M., Salton L. (2011). Biochemistry of Hellp Syndrome. Adv. Clin. Chem..

[3] Alese M.O., Moodley J., Naicker T. (2019). Preeclampsia and Hellp Syndrome, the Role of the Liver. J. Matern. Fetal. Neonatal Med..

[4] Smulian J., Shen-Schwarz S., Scorza W., Kinzler W., Vintzileos A. (2004). A Clinicohistopathologic Comparison between Hellp Syndrome and Severe Preeclampsia. J. Matern. Fetal. Neonatal Med..

[5] Bussen S., Sutterlin M., Steck T. (1999). Plasma Endothelin and Big Endothelin Levels in Women with Severe Preeclampsia or Hellp-Syndrome. Arch. Gynecol. Obstet..

[6] Hulstein J.J., van Runnard Heimel P.J., Franx A., Lenting P.J., Bruinse H.W., Silence K., de Groot P.G., Fijnheer R. (2006). Acute Activation of the Endothelium Results in Increased Levels of Active Von Willebrand Factor in Hemolysis, Elevated Liver Enzymes and Low Platelets (Hellp) Syndrome. J. Thromb. Haemost..

[7] Tranquilli A.L., Landi B., Corradetti A., Giannubilo S.R., Sartini D., Pozzi V., Emanuelli M. (2007). Inflammatory Cytokines Patterns in the Placenta of Pregnancies Complicated by Hellp (Hemolysis, Elevated Liver Enzyme, and Low Platelet) Syndrome. Cytokine.

[8] Khalid F., Tonismae T. (2020). Hellp Syndrome. Statpearls.

[9] Wallace K., Harris S., Addison A., Bean C. (2018). Hellp Syndrome: Pathophysiology and Current Therapies. Curr. Pharm. Biotechnol..

[10] Shen F., Wei J., Snowise S., DeSousa J., Stone P., Viall C., Chen Q., Chamley L. (2014). Trophoblast Debris Extruded from Preeclamptic Placentae Activates Endothelial Cells: A Mechanism by Which the Placenta Communicates with the Maternal Endothelium. Placenta.

[11] Haram K., Svendsen E., Abildgaard U. (2009). The Hellp Syndrome: Clinical Issues and Management. A Review. BMC Pregnancy Childbirth.

[12] Davisson R.L., Hoffmann D.S., Butz G.M., Aldape G., Schlager G., Merrill D.C., Sethi S., Weiss R.M., Bates J.N. (2002). Discovery of a Spontaneous Genetic Mouse Model of Preeclampsia. Hypertension.

[13] Heyward C.Y., Sones J.L., Lob H.E., Yuen L.C., Abbott K.E., Huang W., Begun Z.R., Butler S.D., August A., Leifer C.A. (2017). The Decidua of Preeclamptic-Like Bph/5 Mice Exhibits an Exaggerated Inflammatory Response During Early Pregnancy. J. Reprod. Immunol..

[14] Lacko L.A., Massimiani M., Sones J.L., Hurtado R., Salvi S., Ferrazzani S., Davisson R.L., Campagnolo L., Stuhlmann H. (2014). Novel Expression of Egfl7 in Placental Trophoblast and Endothelial Cells and Its Implication in Preeclampsia. Mech. Dev..

[15] Olson K.N., Reijnders D., Gomes V.C.L., Hebert R.C., Liu C.C., Stephens J.M., Redman L.M., Douglas N.C., Sones J.L. (2020). Complement in Reproductive White Adipose Tissue Characterizes the Obese Preeclamptic-Like Bph/5 Mouse Prior to and During Pregnancy. Biology.

[16] Reijnders D., Liu C.C., Xu X., Zhao A.M., Olson K.N., Butler S.D., Douglas N.C., Sones J.L. (2018). Celecoxib Restores Angiogenic Factor Expression at the Maternal-Fetal Interface in the Bph/5 Mouse Model of Preeclampsia. Physiol. Genom..

[17] Sones J.L., Cha J., Woods A.K., Bartos A., Heyward C.Y., Lob H.E., Isroff C.E., Butler S.D., Shapiro S.E., Dey S.K. (2016). Decidual Cox2 Inhibition Improves Fetal and Maternal Outcomes in a Preeclampsia-Like Mouse Model. JCI Insight.

[18] Sones J.L., Merriam A.A., Seffens A., Brown-Grant D.A., Butler S.D., Zhao A.M., Xu X., Shawber C.J., Grenier J.K., Douglas N.C. (2018). Angiogenic Factor Imbalance Precedes Complement Deposition in Placentae of the Bph/5 Model of Preeclampsia. FASEB J..

[19] Hoffmann D.S., Weydert C.J., Lazartigues E., Kutschke W.J., Kienzle M.F., Leach J.E., Sharma J.A., Sharma R.V., Davisson R.L. (2008). Chronic Tempol Prevents Hypertension, Proteinuria, and Poor Feto-Placental Outcomes in Bph/5 Mouse Model of Preeclampsia. Hypertension.

[20] Woods A.K., Hoffmann D.S., Weydert C.J., Butler S.D., Zhou Y., Sharma R.V., Davisson R.L. (2011). Adenoviral Delivery of Vegf121 Early in Pregnancy Prevents Spontaneous Development of Preeclampsia in Bph/5 Mice. Hypertension.

[21] Reijnders D., Olson K.N., Liu C.C., Beckers K.F., Ghosh S., Redman L.M., Sones J.L. (2019). Dyslipidemia and the Role of Adipose Tissue in Early Pregnancy in the Bph/5 Mouse Model for Preeclampsia. Am. J. Physiol. Regul. Integr. Comp. Physiol..

[22] Sutton E.F., Lob H.E., Song J., Xia Y., Butler S., Liu C.C., Redman L.M., Sones J.L. (2017). Adverse Metabolic Phenotype of Female Offspring Exposed to Preeclampsia in Utero: A Characterization of the Bph/5 Mouse in Postnatal Life. Am. J. Physiol. Regul. Integr. Comp. Physiol..

[23] Dokras A., Hoffmann D.S., Eastvold J.S., Kienzle M.F., Gruman L.M., Kirby P.A., Weiss R.M., Davisson R.L. (2006). Severe Feto-Placental Abnormalities Precede the Onset of Hypertension and Proteinuria in a Mouse Model of Preeclampsia. Biol. Reprod..

[24] Liang W., Menke A.L., Driessen A., Koek G.H., Lindeman J.H., Stoop R., Havekes L.M., Kleemann R., van den Hoek A.M. (2014). Establishment of a General Nafld Scoring System for Rodent Models and Comparison to Human Liver Pathology. PLoS ONE.

[25] Young C.N., Cao X., Guruju M.R., Pierce J.P., Morgan D.A., Wang G., Iadecola C., Mark A.L., Davisson R.L. (2012). Er Stress in the Brain Subfornical Organ Mediates Angiotensin-Dependent Hypertension. J. Clin. Investig..

[26] Livak K.J., Schmittgen T.D. (2001). Analysis of Relative Gene Expression Data Using Real-Time Quantitative Pcr and the 2(-Delta Delta C(T)) Method. Methods.

[27] Oe Y., Ko M., Fushima T., Sato E., Karumanchi S.A., Sato H., Sugawara J., Ito S., Takahashi N. (2018). Hepatic dysfunction and thrombocytopenia induced by excess sFlt1 in mice lacking endothelial nitric oxide synthase. Sci. Rep..

[28] Isler C.M., Bennett W.A., Rinewalt A.N., Cockrell K.L., Martin J.N., Morrison J.C., Granger J.P. (2003). Evaluation of a rat model of preeclampsia for HELLP syndrome characteristics. J. Soc. Gynecol. Investig..

[29] Di Lorenzo A., Bolli E., Tarone L., Cavallo F., Conti L. (2020). Toll-Like Receptor 2 at the Crossroad between Cancer Cells, the Immune System, and the Microbiota. Int. J. Mol. Sci..

[30] Antonelli A., Ferrari S.M., Giuggioli D., Ferrannini E., Ferri C., Fallahi P. (2014). Chemokine (C-X-C Motif) Ligand (Cxcl)10 in Autoimmune Diseases. Autoimmun. Rev..

[31] Sibai B.M. (1990). The Hellp Syndrome (Hemolysis, Elevated Liver Enzymes, and Low Platelets): Much Ado About Nothing?. Am. J. Obstet. Gynecol..

[32] Reubinoff B.E., Schenker J.G. (1991). Hellp Syndrome--a Syndrome of Hemolysis, Elevated Liver Enzymes and Low Platelet Count--Complicating Preeclampsia-Eclampsia. Int. J. Gynaecol. Obstet..

[33] Vinnars M.T., Wijnaendts L.C., Westgren M., Bolte A.C., Papadogiannakis N., Nasiell J. (2008). Severe Preeclampsia with and without Hellp Differ with Regard to Placental Pathology. Hypertension.

[34] Gibbens J., Morris R., Bowles T., Spencer S.K., Wallace K. (2017). Dysregulation of the Fas/Fasl System in an Experimental Animal Model of Hellp Syndrome. Pregnancy Hypertens..

[35] Gibbens J., Spencer S.K., Solis L., Bowles T., Kyle P.B., Szczepanski J.L., Dumas J.P., Robinson R., Wallace K. (2020). Fas Ligand Neutralization Attenuates Hypertension, Endothelin-1, and Placental Inflammation in an Animal Model of Hellp Syndrome. Am. J. Physiol. Regul. Integr. Comp. Physiol..

[36] Trottmann F., Baumann M., Amylidi-Mohr S., Surbek D., Risch L., Mosimann B., Raio L. (2019). Angiogenic Profiling in Hellp Syndrome Cases with or without Hypertension and Proteinuria. Eur. J. Obstet. Gynecol. Reprod. Biol..

